# Converging Mechanisms of Epileptogenesis and Their Insight in Glioblastoma

**DOI:** 10.3389/fnmol.2022.903115

**Published:** 2022-06-27

**Authors:** Kate E. Hills, Kostas Kostarelos, Robert C. Wykes

**Affiliations:** ^1^Nanomedicine Lab, Faculty of Biology, Medicine and Health, University of Manchester, Manchester, United Kingdom; ^2^Catalan Institute for Nanoscience and Nanotechnology (ICN2), Edifici ICN2, Campus UAB, Barcelona, Spain; ^3^Department of Clinical and Experimental Epilepsy, UCL Queen Square Institute of Neurology, London, United Kingdom

**Keywords:** glioma, peritumoral border, epilepsy, seizures, spreading depolarizations

## Abstract

Glioblastoma (GBM) is the most common and advanced form of primary malignant tumor occurring in the adult central nervous system, and it is frequently associated with epilepsy, a debilitating comorbidity. Seizures are observed both pre- and post-surgical resection, indicating that several pathophysiological mechanisms are shared but also prompting questions about how the process of epileptogenesis evolves throughout GBM progression. Molecular mutations commonly seen in primary GBM, i.e., in *PTEN* and p53, and their associated downstream effects are known to influence seizure likelihood. Similarly, various intratumoral mechanisms, such as GBM-induced blood-brain barrier breakdown and glioma-immune cell interactions within the tumor microenvironment are also cited as contributing to network hyperexcitability. Substantial alterations to peri-tumoral glutamate and chloride transporter expressions, as well as widespread dysregulation of GABAergic signaling are known to confer increased epileptogenicity and excitotoxicity. The abnormal characteristics of GBM alter neuronal network function to result in metabolically vulnerable and hyperexcitable peri-tumoral tissue, properties the tumor then exploits to favor its own growth even post-resection. It is evident that there is a complex, dynamic interplay between GBM and epilepsy that promotes the progression of both pathologies. This interaction is only more complicated by the concomitant presence of spreading depolarization (SD). The spontaneous, high-frequency nature of GBM-associated epileptiform activity and SD-associated direct current (DC) shifts require technologies capable of recording brain signals over a wide bandwidth, presenting major challenges for comprehensive electrophysiological investigations. This review will initially provide a detailed examination of the underlying mechanisms that promote network hyperexcitability in GBM. We will then discuss how an investigation of these pathologies from a network level, and utilization of novel electrophysiological tools, will yield a more-effective, clinically-relevant understanding of GBM-related epileptogenesis. Further to this, we will evaluate the clinical relevance of current preclinical research and consider how future therapeutic advancements may impact the bidirectional relationship between GBM, SDs, and seizures.

## Introduction

Glioblastoma (GBM) is an astrocytic-origin neoplasm categorized as a WHO grade IV glioma (Ohgaki and Kleihues, 2013). It is generally regarded as the most aggressive form of primary brain cancer in adults, evidenced by its notoriously high mortality rate. Median survival after primary diagnosis is ~14 months when following standard treatment regimens (Goodwin and Laterra, 2010; Lim et al., 2011; Szopa et al., 2017; Khandwala et al., 2021). Long-term survival is so exceedingly rare that long-term survivor status is achieved only 2.5 years post-diagnosis (Thakkar et al., 2014). The presence and progression of GBM is often associated with seizures, a debilitating comorbidity otherwise known as tumor-associated epilepsy (TAE). Epilepsy associated with GBM is regarded as a “*secondary focal epilepsy*” as it progressively develops secondary to the nature and presence of the primary lesion (de Curtis et al., 2015). The high chance of seizure recurrence in GBM, primarily due to the presence of the neoplasm, means that patients are often diagnosed with and treated for epilepsy following initial seizure occurrence.

Seizures manifest as a presenting symptom in two-thirds of GBM patients, with the average incidence of epilepsy throughout the disease course varying between 30% and 62% (Bruna et al., 2013; Kerkhof and Vecht, 2013; Kerkhof et al., 2013; Dührsen et al., 2019). Seizures in GBM are varied in semiology. The most common types seen in the clinic are focal to bilateral tonic clonic, and either simple or complex focal seizures (Kerkhof et al., 2013; Liang et al., 2016; Toledo et al., 2017). The initial focal onset of the seizures indicates that the transition to seizure is influenced by the location of the tumor, and in general GBM patients with tumors located in cortical regions are at the highest risk of experiencing seizures (Chaichana et al., 2009; Englot et al., 2012; Ertürk Çetin et al., 2017). Lesions situated in the temporal lobe, frontal lobe, and parietal lobe are particularly epileptogenic (Liang et al., 2016). Patients with temporal lobe involvement are thought to be most likely to develop pre-operative seizures due to the high epileptogenicity of mesial temporal lobe structures, as observed in epilepsy syndromes such as temporal lobe epilepsy (TLE). GBMs in cerebellar or brainstem regions are thought to carry a reduced risk of seizures, but these tumors are rare regardless (van Breemen et al., 2007; Lee J. W. et al., 2010; Zhang et al., 2020).

In this review, we will first consider the mechanisms that underlie epileptogenesis in GBM and the impact of their resulting bidirectional relationship on disease progression. We focus on both “intratumoral” and “peri-tumoral” mechanisms that involve epigenetic changes, neuroinflammation, BBB dysfunction, and extensive alterations to glutamatergic and GABAergic neurotransmission. We then recognize in our review that our extensive knowledge gap has implications for clinical translation, and address the challenges faced in current preclinical research. We also then discuss the potential influence of anti-tumorigenic and anti-seizure treatments on GBM-associated epileptogenesis. Finally, we evaluate how approaching these pathologies from a network level is a necessary direction for translational future research.

## The Importance of Timing

There are four main stages associated with clinical GBM progression: pre-resection, post-resection, recurrence, and end-of-life. The occurrence of seizures relative to these defined periods, i.e., their timing, has potential implications for disease progression and prognosis. The occurrence of seizures as a presenting symptom has historically had a more favorable relationship to prognosis, vs. their appearance post-primary diagnosis. This former connection is primarily based on the hypothesis that seizures provoke earlier tumor discovery and initiation of anti-tumorigenic treatment, translating to increased survival. Yet, recent clinical evidence has indicated a significant association between new-onset seizures post-GBM diagnosis and mortality in *IDH-wildtype* GBM patients (Climans et al., 2020; Wasade et al., 2020; Mastall et al., 2021). This disparity suggests that the mechanisms underlying seizure occurrence at these respective time points may slightly differ in their progression. Along the same vein, the recurrence or new-onset occurrence of seizures post-resection is associated with more negative outcomes. In particular, seizure recurrence following first-line anti-tumor therapy and successful seizure control is closely associated with disease progression (Chaichana et al., 2009; Vecht et al., 2014).

**Figure 1:**
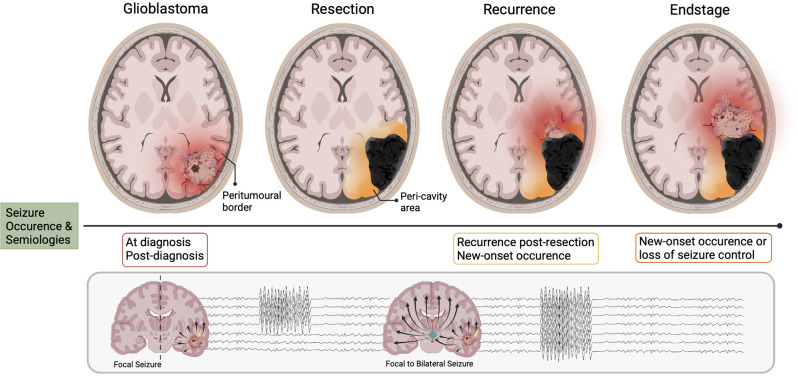
Seizure occurrence and pathoanatomical changes relative to disease stages in glioblastoma. The different stages of glioblastoma progression are associated with their own relationship to seizure occurrence, of which focal and focal-to-bilateral are the most common semiology. When the bulk tumor is present (glioblastoma), the area where tumor meets “normal brain” is known as the peri-tumoral border (in red). The network organization of this area changes progressively with disease evolution. Upon resection, the peri-tumoral border is now termed the peri-cavity area. Now independent of the glioblastoma, this area may become intrinsically epileptic. When the tumor recurs, areas of the peri-cavity area now merge with the new peri-tumoral border (orange/red). At the endstage of the disease, the peri-cavity area and peri-tumoral border are simultaneously present (orange/red). Therefore, there are a multitude of mechanisms generating seizure activity both in the new peri-tumoral border and the epileptic peri-cavity area.

It is commonplace that patients with pre-operative seizures will experience seizure re-emergence post-resection. If GBM is considered the singular “driving force” of seizure generation then it is reasonable to suggest that removal of the bulk tumor should result in complete seizure cessation. However, this is more often not the case. Those with lesions previously situated in the frontal lobe carry the highest risk of post-operative seizures; a switch with temporal lobe lesions, which are associated with the highest pre-operative seizure likelihood (Liang et al., 2016). Interestingly, patients with no previous history of TAE have also been known to develop new-onset seizures post-tumor resection. Liang et al. (2016) found that 45% of their GBM cohort without any prior seizure history developed new-onset epilepsy 12 months post-resection. It is well established that epileptogenesis is an ongoing process that continues after seizure onset, and in the case of GBM, this process takes place in the peri-tumoral brain tissue as opposed to the tumor itself (Figure 1; Köhling et al., 2006). Seizure generation may continue or newly arise post-tumor removal, as the epileptogenic loci is often still present in the remaining “normal” parenchyma surrounding the resection cavity, otherwise known as the peri-cavity area. Furthermore, the infiltrative nature of GBM confers that small populations of tumor cells will be present in the peri-tumoral border post-resection, and these may continue to promote the cycle of epileptogenesis. The influence of tumor resection on, and the potential post-resection mechanisms underlying seizure generation will be discussed further in-depth later in our surgical resection and recurrence section.

The disease’s end-of-life phase often includes the development of new-onset seizures in patients with no history of TAE, or recurrence of seizures in patients with previously effective anti-epileptic regimens (Oberndorfer et al., 2008; Pace et al., 2013; Koekkoek et al., 2014). The non-linear appearance of seizures with GBM progression suggests that the mechanisms by which seizures manifest prior to, or following diagnosis (pre-surgical resection) may be different to those resulting in post-resection recurrent or new-onset seizures. And these may be different still from those occurring in the final phase of the disease. The presence of seizures post-resection infers that whilst GBM appears to initially influence the development of epilepsy, the mechanisms by which seizures are continuously generated may eventually become independent of the tumor.

The importance of seizure occurrence relative to disease stage has only recently been delineated, and this reflects the historical inconsistencies within the literature when reporting seizure occurrence in GBM. The extent of the relationship between seizures and prognosis has been clouded by studies grouping GBM-associated seizures in investigations of low-grade gliomas. This is exacerbated by the lack of distinction between *IDH-mutant* vs. *IDH-wildtype* tumors in studies. It is evident that the mechanisms underlying network hyperexcitability, and their effect on disease progression, are extremely varied between tumors despite the umbrella classification of “glioma”. The inclusion of GBM-associated seizures in studies of lower-grade gliomas leads to misinterpretation of the role seizures play in GBM progression. Further to this, the *IDH* subtypes of GBM show distinct relationships to seizure occurrence and prognosis. Higher rates of seizures are often associated with the *IDH-mutant* GBMs that are known to occur in younger patients, evolve from lower-grade tumors, and have a more favorable prognosis (Alzial et al., 2022). Accordingly, the mechanisms contributing to seizure generation in this patient population (*IDH-mutant*) are thought to be markedly different to those occurring in *IDH-wildtype* patients (Liubinas et al., 2014; Berendsen et al., 2016; Toledo et al., 2017; Wasade et al., 2020). Therefore, it is crucial that future studies differentiate not only between low and high-grade gliomas but also subtypes.

## Factors Contributing to Tumor-Driven Epileptogenesis

### Epileptogenic Molecular Mutations

The rapid growth of GBM and the presence of areas of hemorrhage or necrosis confers that there is a high probability of acute tissue damage, the consequences of which could promote epileptogenesis. Yet, clinical studies have not observed a strong correlation between tumor volume, seizures, and mass effect. Generally, smaller tumor volume is associated with seizure presence, and so other tumor-driven mechanisms may provoke epileptogenesis (Chaichana et al., 2009; Henker et al., 2019). GBM’s disorganized, uncontrolled growth inevitably leads to mutations, and consequently, there is substantial genetic heterogeneity inter- and even intratumorally (Szopa et al., 2017). Collective mutations in tumor suppressor genes *PTEN* (Wang et al., 1997; Han et al., 2016), *TP53* (Dittmer et al., 1993; Nagpal et al., 2006; Zhang et al., 2018), and *NF1* (Pearson and Regad, 2017; Soomro et al., 2017; Szopa et al., 2017) are common in primary GBM. Disruptions to their associated downstream signaling pathways are heavily implicated in tumorigenesis. Interestingly, *PTEN*, *NF1*, and *TP53* are all also intrinsically linked to epilepsy. Recent experimental studies have selected them for deletion/mutation to produce genetic rodent models of GBM that reliably produce epileptogenic tumors (Hatcher et al., 2020; Hu et al., 2022). Their commonality in both GBM and epilepsy indicates that whilst the tumor itself may not be electrographically active, its intrinsic molecular properties produce an environment that is both vulnerable to and promotes mechanisms of hyperexcitability.

Physiologically, *PTEN* negatively regulates PI3K/AKT/mTOR pathway. However, mutated or deleted forms of *PTEN* are present in GBM resulting in the disinhibition of PI3K/AKT and hyperactivation of mTOR signaling (Endersby and Baker, 2008; Venkatesan et al., 2016). *PTEN* mutations have been associated with poorer prognosis in GBM and are also known to play a role in epilepsy syndromes. Targeted conditional deletion of *PTEN* in mice results in spontaneous seizures with post-mortem brains displaying the hallmark pathologies of temporal lobe epilepsy (Nishio et al., 2000; Backman et al., 2001; Luikart et al., 2011; Pun et al., 2012; Williams et al., 2015). *NF1*, and its protein neurofibromin 1, are known to negatively regulate Ras signaling through GTPase activity (Pearson and Regad, 2017; Soomro et al., 2017). GBM-induced loss-of-function mutations in *NF1* disinhibits the Ras/MAPK pathway, leading to hyperactivation of mTOR and unchecked cell proliferation, favoring tumor progression. Targeted knockout of the *NF1* gene is associated with decreased latency to epilepsy and greater seizure severity in mice (Sabetghadam et al., 2020). Its mutated form is associated with increased seizure presence in patients with neurofibromatosis-1 (Sorrentino et al., 2021). Mutations in *TP53*, also known as p53, disrupt its ability to induce the mechanisms of cell cycle arrest, senescence, and apoptosis that prevent proliferation and migration of damaged or neoplastic cells (Kastenhuber and Lowe, 2017). Gain-of-function (GOF) mut-p53 is a protein highly expressed in GBM that enhances pro-invasive signaling by upregulating the activity of receptor tyrosine kinases e.g., MET and EGFR (Zhang et al., 2018). Increased expression of p53, particularly within the hippocampus, is found in both experimental models and resected tissue samples from patients with drug-resistant TLE (Morrison et al., 1996; Engel et al., 2007). In seizures, overexpression of p53 is associated with greater activation of apoptotic processes and neuronal cell death, further contributing to network imbalances and potentiating mechanisms of excitability (Morrison et al., 1996). Importantly, elevated p53 expression has been previously correlated with epileptogenic GBMs (Toledo et al., 2017). It is worth noting that *TP53* mutations alone are not sufficient to induce the formation of GBM, and require other genes such as *PTEN* to also be mutated to drive GBM progression (Zheng et al., 2008).

Whilst there are overt epileptogenic molecular mutations potentiated by GBM growth as discussed above, the epileptogenic potential of a tumor may also be influenced by the particular subpopulation of glioma cells that constitute the tumor. An interesting investigation into this relationship is seen in Lin et al. (2017). They utilized their CRISPR-Cas9 in-utero electroporation (IUE) GBM model to identify glioma-analogs of astrocyte subpopulations specifically involved in the emergence of GBM-associated seizures. One such identified was astrocyte population C which increased linearly with tumor progression. This trend was tightly correlated with increased invasion and importantly, seizure onset. Interestingly, astrocyte population C was found to be highly enriched for the expression of synaptic genes, and also for a number of established epilepsy-associated genes. This indicates an additional genetic glial basis for epileptogenesis in GBM. These findings also confer that some of the most intrinsic properties of the tumor, the genes enriching the glioma cells, directly influence the development of epilepsy.

### BBB Dysfunction

GBM is one of the most vascularized tumors in humans and its associated neoangiogenesis promotes blood-brain barrier (BBB) disruption. This breakdown allows infiltration of serum proteins into the brain parenchyma, encourages vasogenic oedema, and increases intracranial pressure (Noell et al., 2012). Pathologic BBB disruption is readily apparent in GBM patients using gadolinium contrast MRI, whereby the leaky BBB enables gadolinium to diffuse into the tissue and presents as ring-enhancing lesions (Wolburg et al., 2012; Dubois et al., 2014). Morphologically, blood vessels involved in the GBM blood-tumor barrier are typically non-uniform with altered pericyte coverage, fenestrations, and reduced tight junctions. For in-depth reviews focused on tumor progression and its impact on the BBB, please see Dubois et al. (2014) and Arvanitis et al. (2020). It is notable that BBB dysfunction is observed in both human and animal studies following acute seizures, and its leakage is particularly associated with TLE (van Vliet et al., 2007; Marchi et al., 2012). As a corollary, there may be additional effects on the BBB in GBM due to the effect of epileptogenesis on pericytes, please see Löscher and Friedman (2020) and Yamanaka et al. (2021) for further discussion of this work. Interestingly, epileptogenesis secondary to BBB injury largely involves the activation of inflammatory responses and reactive astrogliosis, attributes observed in the GBM microenvironment.

The exposure of the brain to blood serum components such as fibrinogen, albumin, glutamate, and K^+^ following GBM-induced BBB disruption contributes to changes in neuronal excitability. The transformation of astrocytes under these conditions further provokes epileptogenic changes. The action of albumin at transforming growth factor beta (TGF-β) receptors induces changes in the expression of critical inwardly rectifying potassium channels (Kir; Ivens et al., 2007; Marchi et al., 2012) and water channels i.e., aquaporin 4 (AQP4; Ikeshima-Kataoka, 2016). In human and animal models of epilepsy syndromes, it has been shown that serum-exposure can lead to the direct downregulation or mislocalization of Kir4.1 astrocytic K^+^ channels consequently resulting in disrupted K^+^ homeostasis (Campbell et al., 2012; Buckingham and Robel, 2013; Pallud et al., 2013). Kir4.1 is responsible for buffering extracellular K^+^ and maintaining a negative resting membrane potential (RMP; Olsen and Sontheimer, 2004; Hibino et al., 2010). Therefore, GBM-induced disruption would likely result in RMP dysregulation of astrocyte processes, accumulation of extracellular K^+^ and consequently a lowered neuronal firing threshold (Table 1; Olsen and Sontheimer, 2004). GBM-induced AQP4 dysregulation and redistribution is known to be associated with increased peri-tumoral vasogenic oedema and aberrant BBB function (Warth et al., 2007).

**Table 1:** Potential biomarkers in GBM-associated epilepsy, their relationship to each pathology, role, and potential targeted therapies.

**Potential biomarkers**	**Associated cell types**	**Alterations in GBM (G) and epileptogenesis (E)**	**Pathological role**	**Potential targeted therapies**	**References**
**Kir4.1**	Astrocytes	G and E: Downregulation and mislocalization	Disrupts astrocytic RMP → extracellular K^+^ accumulation; suppression of glutamate reuptake.	Gene therapy, VPA (CA)	Mukai et al. (2018) and Ohno et al. (2021)
**Aquaporin 4**	Astrocytes, glioma cells	G: Upregulated with diffuse perivascular expression E: Extremes of expression, mislocalization	Cell swelling → efflux of K^+^, Cl^−^, glutamate; decreased ECS volume.	Gene therapy, acetazolamide (CA)	Reiss and Oles (1996); Binder et al. (2004a); Alvestad et al. (2013); and Duan and Di (2017)
**MMP-9**	Astrocytes, glioma cells	G and E: Increased release	Increases NMDAR activity; degrades PNNs surrounding FS-PV+ interneurons.	MMP-inhibitors (e.g., marimastat)	Bronisz and Kurkowska-Jastrzębska (2016); Mondal et al. (2020); and Pijet et al. (2020)
**xCT (SLC7A11)**	Astrocytes, glioma cells	G and E: Upregulation	Increases glutamate release into the extracellular space.	Gene therapy, sulfasalazine (CA)	Lewerenz et al. (2014); Leclercq et al. (2019); and Sears et al. (2019)
**GLT-1**	Astrocytes	G and E Marked downregulation on astrocytes	Impairs glutamate clearance from the extracellular space.	Gene therapy, ceftriaxone (CA)	de Groot et al. (2005); Peterson et al. (2021); and Ramandi et al. (2021)
**GABA_A_R**	Pyramidal neurons, glioma cells	G: Almost complete absence on glioma cells E: Dysfunctional membrane trafficking, various subunit alterations	Reduces GABAergic neurotransmission → glioma cell proliferation; disinhibits pyramidal neuronal firing	Gene therapy, benzodiazepines (CA)	Houser et al. (2012) and Greenfield (2013)
**NKCC1:KCC2**	Pyramidal neurons	G and E: NKCC1 upregulated on glioma cells in GBM and pyramidal cells, KCC2 downregulated on pyramidal cells	Reverses the chloride gradient → paradoxical depolarization; cell shrinkage promoting glioma cell proliferation	NKCC1: Bumetanide (CA) KCC2: Gene therapy, kenpaullone (CA)	Huberfeld et al. (2007); MacKenzie and Maguire (2015); MacKenzie et al. (2016); Duy et al. (2020); and Yeo et al. (2021)

*CA, clinically approved for use in epilepsy or otherwise; VPA, valproic acid*.

### Ion Homeostasis

Notably, Kir4.1-mediated K^+^ buffering also plays a crucial role in glutamate homeostasis as the channel tends to colocalize with GLT-1 and AQP4 at tripartite synapses (Nagelhus et al., 2004; Olsen and Sontheimer, 2008). Glutamate reuptake is suppressed in the case of Kir4.1 inhibition, due to disruption of ionic gradients and astrocytic RMP, with epileptogenic consequences. These occur secondary to greater extracellular glutamate accumulation, as will be discussed later in glutamate release, reuptake, and post-synaptic action (Kucheryavykh et al., 2007; Armbruster et al., 2021). Both downregulation and mislocalization of Kir4.1 has been demonstrated in patient-derived GBM samples and cell lines (Zurolo et al., 2012; Madadi et al., 2021). As well as significant electrophysiological changes including a depolarized astrocytic RMP and large outward K^+^ currents (Olsen and Sontheimer, 2004).

**Figure 2:**
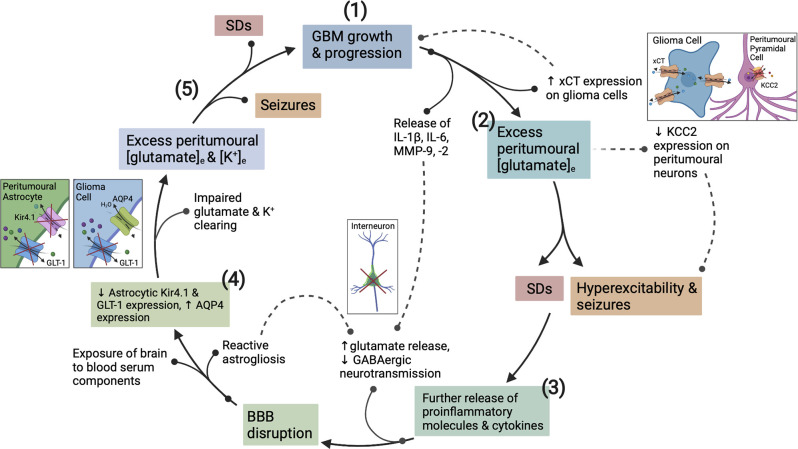
The vicious cycle of hyperexcitability in glioblastoma (GBM) progression. How epileptogenesis is initiated, is influenced by, and contributes to GBM growth. **(1)** GBM growth and its interaction with neuronal/glial cells initiates the cycle to epileptogenesis. **(2)** Upregulation of xCT on glioma cells and downregulation of KCC2 on surrounding pyramidal neurons creates an initial imbalance between excitatory and inhibitory neurotransmission. **(3)** Pro-inflammatory cytokines and MMPs present in the GBM TME act to degrade the PNNs surrounding GABAergic interneurons. **(3)** Once epileptogenesis has been initiated, further proinflammatory cytokine, K^+^, and glutamate release exacerbates BBB disruption and encourages network reorganization creating a microenvironment conducive to SDs. **(4)** The function of Kir4.1, AQP4 and GLT-1 transporters is disrupted by recurrent seizures and SDs, and *via* actions of GBM. **(5)** This dysfunction continues in a cyclical fashion whereby seizures promote more seizures and SDs, and the associated pathological downstream signaling actively potentiates glioma cell proliferation, and *vice versa*. BBB, blood brain-barrier; TME, tumor microenvironment; MMPs, matrix metalloproteinases; PNNs, perineuronal nets; SDs, spreading depolarizations; APQ4, aquaporin 4.

Accumulation of extracellular K^+^ is known to be a key initiating event in the generation of spreading depolarization (SD). SD is characterized by a near-complete regional depolarization of neurons and glial cells that is accompanied by the collapse of transmembrane ion gradients and followed by a temporary suppression of neuronal activity (Pietrobon and Moskowitz, 2014; Ayata and Lauritzen, 2015). SDs are primarily self-terminating in healthy brain tissue as the resources required to recover from major disruptions to transmembrane ion gradients are readily available. However, in a metabolically compromised brain, such as one with GBM, there is decreased availability of these resources. Consequently, repolarization and recovery is significantly delayed to a point that may exacerbate existing tissue damage (Ayata and Lauritzen, 2015). The complex relationship between seizures and SDs is well documented, seizures create an excitotoxic environment conducive to SD generation and *vice versa* (Berger et al., 2008; Kramer et al., 2017). The BBB disruption and subsequent steady increase in K^+^ accumulation that occurs within the GBM peri-tumoral border contributes to a progressive reduction in the threshold for seizures and SDs (Figure 2).

Upregulation and redistribution of AQP4 is widely reported in GBM (Figure 2). Interestingly significantly increased diffuse and perivascular expression of AQP4 on glioma cells is seen in resected tumor tissue from GBM patients with seizures vs. those without (Isoardo et al., 2012). Dysregulation of astrocytic AQP4 expression is known to contribute to epileptogenesis in traditional epilepsy syndromes (Binder and Steinhäuser, 2006; Binder et al., 2012). AQP4-knockout mice demonstrate increased latency to seizure generation when challenged with pentylenetetrazole (PTZ). This is largely attributed to expansion of the extracellular space (ECS), but seizures showed a more severe phenotype once eventually generated (Binder et al., 2004a, 2006). Conversely, significant AQP4 upregulation is observed in TLE and in the chronically epileptic brain following intra-hippocampal kainic acid (IHKA) epilepsy induction (Lee et al., 2004; Hubbard et al., 2016). This suggests that extremes of AQP4 expression contribute to epileptogenesis (Binder et al., 2004a, b). GBM-induced upregulation of AQP4 expression may lead to cell swelling, compensatory efflux of K^+^, Cl^−^, and glutamate, and decreased ECS volume (Table 1). The net effect of which would favor neuronal depolarization and potentiation of excitatory activity throughout the surrounding neuronal network (Binder et al., 2012). Therefore, GBM-induced alterations to both AQP4 and Kir4.1 aggressively promote microenvironment changes conducive to both seizures and SDs.

### Neuroinflammation

The extreme infiltrative and epileptogenic nature of GBM can be, at least in part, attributed to its master manipulation of the immune system. GBMs are primarily immunosuppressive tumors, and this is primarily due to the high presence of tumor-associated macrophages (TAMs), tumor-associated neutrophils (TANs), and regulatory T cells (Tregs). These mediators are crucial in maintaining GBM’s extreme immunosuppressive phenotype and are also directly linked to cancer cell proliferation and the recruitment of surrounding non-neoplastic cells (Fanelli et al., 2021). However, whilst the net effect of the tumor microenvironment (TME) is immunosuppressive, this does not preclude the existence of underlying pro-inflammatory actions. The peri-tumoral environment is inundated with a variety of pro-inflammatory cytokines (IL-1β, IL-6, IL-8, TNF-α), chemokines, and extracellular matrix remodeling enzymes (MMP-2, MMP-9; Figure 2; DeCordova et al., 2020). Reactive astrocytes, which also produce said mediators (Guan et al., 2018), are also increasingly present. In particular, IL-1β and IL-6 are well recognized to contribute to oncogenesis and their expression is abundantly found in GBM cell lines and tissues (Lu et al., 2007; Yeung et al., 2012; Liu et al., 2021), IL-6, in particular, is associated with poorer prognosis (Hori et al., 2019). IL-1β signaling stimulates activation of NF-kB, ERK, and p38 MAPK pathway signaling, as well as promoting the release of IL-6 and IL-8 from GBM cells (Yeung et al., 2013). In turn, the release of IL-8 is postulated to induce TNF-α secretion from TAMs (Wei et al., 2021).

Interestingly, the pro-inflammatory molecules seen within the TME are historically associated with seizures (Rana and Musto, 2018). It is well recognized that neuroinflammation and associated BBB breakdown are necessary for epileptogenesis (Marchi et al., 2007). Experimental models of TLE, and resected hippocampal tissue from TLE patients both display upregulated expressions of pro-inflammatory molecules, reactive astrocytosis, and activated microglia (Ravizza et al., 2008). Transgenic mice overexpressing IL-6 and TNF-α demonstrate increased seizure susceptibility (Campbell et al., 1993; Probert et al., 1997; Samland et al., 2003), and treatment with IL-1β prior to kainic acid administration potentiates seizure duration (Vezzani et al., 1999). IL-1β exerts its pro-convulsive actions through IL-1R (IL-1 receptor)-mediated phosphorylation of the NMDAR GluN2B subunit (Viviani et al., 2003). TNF-α upregulates Ca^2+^-permeable AMPAR (CP-AMPAR) membrane expression and trafficking, enabling greater Ca^2+^ influx with cytotoxic effects. It also acts to increase the internalization of GABA_A_ receptors (GABA_A_Rs), subsequently reducing the influence of GABAergic inhibitory neurotransmission (Stellwagen et al., 2005). IL-1β and TNF-α are also known to increase glutaminase activity (Bezzi et al., 2001; Ye et al., 2013), which results in enhanced glutamate release whilst also decreasing its re-uptake (Hu et al., 2000; Yeung et al., 2013; Rana and Musto, 2018). All alterations to network function that can be interpreted as epileptogenic.

Concomitant to pro-inflammatory cytokine secretion, GBM cells release matrix metalloproteinase 9 (MMP-9). Chronic MMP-9 production is associated with BBB disruption, independent of the insult, and mediates its pathological effects by decreasing the efficiency of BBB tight junctions (Asahi et al., 2001). MMP-9 also impacts both excitatory and inhibitory neurotransmission. It acts to increase the activity of NMDARs through integrin-β1 signaling (Michaluk et al., 2009), and induces the degradation of perineuronal nets (PNNs) that surround fast-spiking parvalbumin positive (FS-PV+) inhibitory interneurons (Figure 2; Tewari et al., 2018). These alterations increase the excitability of the peri-tumoral network, and when occurring in conjunction with the epileptogenic mechanisms mentioned in the following section, result in seizure generation. These are just a few mechanisms by which GBM promotes network hyperexcitability to the point of recurrent seizure generation. Following which the seizures themselves potentiate tumor progression and increased excitability in a malignant feed-forward nature.

## Seizures, Spreading Depolarizations and Peri-Tumoral Border

### Glutamate Release, Reuptake and Post-synaptic Action

In the GBM peri-tumoral environment, there is a breakdown in glutamate homeostasis and subsequent pathological network signaling creates an environment favorable to seizures and SDs. In GBM, glutamate is primarily released into the synaptic cleft by the cystine/glutamate antiporter system (system xc-), also known by its active subunit xCT (Buckingham et al., 2011). System xc- primarily mediates the exchange of intracellular L-glutamate for extracellular L-cystine, a precursor for the antioxidant glutathione (GSH). Its expression is known to be significantly upregulated in the tumor tissue of epileptic GBM patients (Lin et al., 2020). Upregulated expression of system xc- in GBM cells leads to greater concentrations of glutamate in the extracellular space (Table 1). This promotes hyperexcitability through activation of post-synaptic glutamatergic receptors on peri-tumoral neurons, subsequently initiating processes of depolarization and excitotoxicity (Figure 2; Buckingham et al., 2011; Neal et al., 2016). As a corollary, increased xCT expression has been associated with shorter progression-free survival and poorer overall survival in GBM patients, as well as a more infiltrative phenotype on MRI (Takeuchi et al., 2013). Interestingly, the expression of xCT in glioma patients has been found to significantly correlate with epileptic seizures at GBM diagnosis, even denoted as an “independent biomarker” (Sørensen et al., 2018). Notably, epileptiform activity can be reduced by treatment of both epileptogenic GBM-bearing acute brain slices and mice with sulfasalazine (SAS), an FDA-approved system xc- antagonist (Table 1; Buckingham et al., 2011; Campbell et al., 2012; Robert et al., 2015; Hatcher et al., 2020). There are no current clinical trials looking at SAS as a treatment for GBM-related epilepsy. Yet, a phase 2 randomized clinical study evaluating SAS as a primary anti-tumor treatment in recurrent GBM was conducted 2005–2007. This trial was terminated early due to unreasonable occurrence of adverse events and lack of tumor response. However, this study was conducted in a severely ill, neurologically impaired patient population. They also neglected to assess the anti-epileptic potential of SAS as an adjuvant treatment to standard anti-tumor regimens and therefore this avenue would still be of great interest (Robe et al., 2009).

The disruption in peri-tumoral glutamate homeostasis is thought to be further exacerbated by GBM-induced downregulation of astrocytic glutamate transporter 1 (GLT-1; Table 1). This impairs clearance of glutamate from the synaptic cleft and indirectly acts to potentiate its actions at post-synaptic NMDARs/AMPARs (Robert and Sontheimer, 2014). GLT-1 (also known as EAAT2) is responsible for 80%–90% of all extracellular glutamate reuptake activity (Lin et al., 2012; Takahashi et al., 2015), but both GBM tumor tissue and cell lines show an almost complete absence of the transporter (Ye et al., 1999; de Groot et al., 2005). Initial experimental evidence suggests that GLT-1 expression is significantly downregulated in both tumoral and peri-tumoral tissue from GBM patients with seizures vs. those without (Yuen et al., 2012). Interestingly, parallels are seen in TLE patients with hippocampal sclerosis and drug-refractory seizure phenotypes where GLT-1 expression is markedly reduced (Proper et al., 2002; Sarac et al., 2009). Additionally, GLT-1 knockout mice are known to develop lethal spontaneous seizures (Tanaka et al., 1997), and GLT-1 conditional knock-out mice demonstrate increased susceptibility to SD (Aizawa et al., 2020). A reduction in GLT-1 expression favors SD propagation by allowing a more rapid increase in extracellular glutamate concentration during the course of the phenomenon (Figure 2). Direct links are yet to be fully elucidated in the literature, but current findings support the theory that the pathological mechanisms underlying seizures and SDs may be in part initiated by GBM-induced GLT-1 downregulation.

The pathological downstream signaling cascades that occur secondary to extracellular glutamate accumulation in GBM are primarily mediated by post-synaptic NMDARs and AMPARs. Glutamate-mediated prolonged NMDAR and CP-AMPAR activation triggers a wide variety of intracellular events, that in turn initiate processes of Ca^2+^-mediated excitotoxicity and apoptosis (Zhu et al., 2000; Buckingham and Robel, 2013; Henley and Wilkinson, 2016). In both peri-tumoral human and murine GBM tissue, there is increased phosphorylation of the NMDAR GluN2B subunit, a widely used measure of NMDAR activation (Gao et al., 2014). Notably, a relative increase in the NMDAR GluN2B subunit confers slower receptor decay times enabling a greater volume of Ca^2+^ influx through the receptor, thereby increasing the risk of Ca^2+^-mediated excitotoxicity and neuronal death. NMDARs are also implicated in the propagation of SDs, therefore any alterations to NMDAR activation may potentiate SD propagation and alter their waveform (Masvidal-Codina et al., 2021).

There are marked decreases in the editing of the AMPAR GluA2 subunit and increases in the expression of GluA1 subunits in human GBM samples and cell lines (Maas et al., 2001; de Groot et al., 2008; Venkataramani et al., 2019). This indicates that there is Ca^2+^ influx through, and increased activity at, post-synaptic ionotropic glutamate receptors that trends towards increased neuronal excitability. Epileptiform activity originating from GBM-bearing tissue can be completely abolished by the administration of NMDAR antagonist APV (Buckingham et al., 2011; Campbell et al., 2012), and AMPAR antagonists perampanel and cyanquixaline (CNQX; Venkataramani et al., 2019; Venkatesh et al., 2019; Lange et al., 2020; Mayer et al., 2020). When occurring in GBM, the surrounding cell death occurring secondary to overactivation of NMDARs/AMPARs could encourage greater tumor growth by “making space” and further exacerbate the network imbalance fuelling excitability.

The generation of seizures in peri-tumoral neuronal networks inevitably disrupts ionic homeostasis. This primarily manifests as increased extracellular K^+^ concentration and intracellular Na^+^, Cl^−^, and Ca^2+^ concentrations, as well as a decrease in intracellular pH (Raimondo et al., 2015). Moreover, recurrent seizures provoke excess glutamate release from pre-synaptic neurons. Parallels can be drawn between the signaling pathways initiated by seizures/SDs and those potentiated by GBM. The dysfunctional actions of glutamate transporters and receptors also create an environment conducive to glioma cell proliferation, migration, and invasion. Blockade of xCT with SAS consistently reduces GBM glioma cell migration in multiple cell lines, an anti-tumor effect corroborated *in vivo* (Lyons et al., 2007). GLT-1 downregulation also contributes to GBM oncogenesis, in physiological conditions it plays a role in growth suppression therefore the absence of this transporter on glioma cells allows their growth to proceed unchecked. Adeno-associated virus (AAV)-mediated reintroduction of GLT-1 into GBM cells mitigated *in vitro* growth. Furthermore, flank implantation of U87 cells transfected *ex vivo* with AAV-GLT-1 into nude mice was associated with significantly decreased tumor growth (de Groot et al., 2005). As such, evidence suggests that glutamate released by GBM glioma cells may be able to promote pro-migration/invasion and proliferative effects through seizure-induced alterations to the functionality of GLT-1, NMDARs, and AMPARs.

### GABAergic Neurotransmission and Chloride Homeostasis

GBM induces major perturbations to inhibitory GABAergic neurotransmission. These disruptions originate from a loss of GABAergic synaptic density on pyramidal neurons, reductions in GABA_A_R expression, and changes to the Cl^−^ gradient. GBM-induced degradation of the PNNs surrounding FS-PV+ inhibitory interneurons significantly decreases their frequency of action potential firing (Figure 2; Tewari et al., 2018). Significant selective loss of FS-PV+ interneurons is also observed within the peri-tumoral cortex, secondary to excitoxicity associated with increased extracellular glutamate accumulation (Tewari et al., 2018). Cumulatively, this manifests as reduced GABAergic neurotransmission and disinhibition of pyramidal neurons within the peri-tumoral network. GBM also induces a reduction in the density of GABA binding sites, such that functional GABA_A_Rs are postulated to be almost completely absent on glioma cells (Labrakakis et al., 1998; Smits et al., 2012; Jung et al., 2019). Experimental studies have demonstrated that the majority of GBM cells, from both human GBM samples and cell lines, do not exhibit any electrophysiological response when challenged with the application of GABA. This is suggestive of a lack of functional GABA receptors (Labrakakis et al., 1998). Whilst glioma cells appear to lack functional GABA_A_Rs, they are still postulated to be present on surrounding peri-tumoral neurons allowing them to play a role in epileptogenic neurotransmission (Campbell et al., 2015; Huberfeld and Vecht, 2016).

GBM-induced alterations to the expression ratio of Cl^−^ transporters KCC2 and NKCC1 influence GABAergic neurotransmission by extruding and accumulating intracellular Cl^−^ ([Cl^−^]_i_), respectively. They are primarily responsible for the pathological reversal of the Cl^−^ gradient that occurs in GBM (Liu et al., 2020; Zhang et al., 2021). Higher KCC2 expression ensures a low [Cl^−^]_i_ concentration such that GABA binding elicits Cl^−^ influx and cell hyperpolarization, potentiating inhibitory GABAergic signaling. Whereas increased NKCC1 expression in mature neurons results in a greater concentration of [Cl^−^]_i_ and reversal of the chloride gradient, promoting Cl^−^ efflux upon GABA binding and cell depolarization (Plotkin et al., 1997; Hübner et al., 2001). Various epilepsy syndromes commonly demonstrate alterations in both Cl^−^ cotransporters (Liu et al., 2020). Significant downregulation of KCC2 is known to occur within the GBM peri-tumoral cortex (Figure 2), correlating with greater [Cl^−^]_i_ concentration and a more positive GABA equilibrium potential (Conti et al., 2011; Pallud et al., 2014; MacKenzie et al., 2016). This has also been shown to significantly correlate with decreased survival in GBM patients (Campbell et al., 2015). Peri-tumoral tissues from patients with epileptogenic GBMs show significantly increased expression of NKCC1 vs. non-epileptogenic patients (Conti et al., 2011; Garzon-Muvdi et al., 2012; Pallud et al., 2014; Schiapparelli et al., 2017; Luo et al., 2020). Epileptic discharges from resected human tissue were suppressed by the application of FDA-approved NKCC1 antagonist bumetanide (Table 1; Pallud et al., 2014). Further to this, latency to seizure onset was increased in GBM-bearing mice treated with continuous infusion of bumetanide (MacKenzie et al., 2016). Evidently, the Cl^−^ dysregulation and consequent defective GABAergic signaling in GBM facilitates the generation of epileptiform activity. GBM-induced changes in Cl^−^ transporter expression may be potentially triggered by overstimulation of NMDARs and consequent dephosphorylation of KCC2 residue serine 940 (Ser940); a residue responsible for transporter function and stability in the membrane (Lee H. H. C. et al., 2010; Lee et al., 2011; MacKenzie et al., 2016). Blockade of excess glutamate release from glioma cells with SAS prevents KCC2 downregulation in GBM cells (MacKenzie et al., 2016), implicating increased glutamate signaling in Cl^−^ gradient dysregulation and aberrant GABAergic neurotransmission. Additionally, BDNF release from glioma cells or locally activated microglia is known to elicit the downregulation of KCC2 and upregulation of NKCC1 on peri-tumoral neurons (Pallud et al., 2013).

Defective GABAergic neurotransmission also confers a growth advantage in GBM. Increased GABAergic signaling *via* GABA_A_Rs has been shown to negatively regulate glioma cell proliferation (Blanchart et al., 2017). Therefore, the observed reduction in functional GABA_A_Rs expression on glioma cells would result in decreased GABAergic neurotransmission and favor tumor growth (Table 1; Labrakakis et al., 1998). NKCC1 is also known to regulate glioma cell volume changes by increasing [Cl^−^]_i_, which results in Cl^−^ efflux upon GABA binding and cell shrinkage to promote glioma cell proliferation, migration, and invasion of surrounding parenchyma (Table 1; Schiapparelli et al., 2017). Both knockdown of NKCC1 and inhibition with bumetanide significantly reduces GBM cell migration *in vitro* and *in vivo*, and overexpression of the transporter markedly increases GBM cell invasion (Haas and Sontheimer, 2010; Garzon-Muvdi et al., 2012; Schiapparelli et al., 2017).

## From Laboratory to Clinical Translation

### Clinical Relevance

The majority of current GBM literature focuses on either the complex GBM microenvironment or the development of novel therapeutics. Ergo, studies on the epileptological aspect of GBM are extremely limited, and even less have investigated the impact of anti-tumorigenic treatment strategies on the relationships between epilepsy, SDs, and tumor growth. Experimental studies of GBM-associated epilepsy primarily employ GBM cell lines (human, mouse, or rat) or human primary cultures to generate intracranial GBM tumors. Wherein a tumor cell suspension is intracranially injected into brain areas associated with seizure vulnerability and/or propagation (Table 2). Genetic manipulation of epilepsy-associated known oncogenes *via* IUE CRISPR/Cas9-mediated deletion or utilization of inducible-Cre-LoxP transgenic models has recently been employed to aid the investigation into genetic mechanisms of GBM-associated epileptogenesis (Table 2; Hatcher et al., 2020; Jin et al., 2021). The majority of existing studies utilize transplantation methods (Köhling et al., 2006; Bouckaert et al., 2020), with many seminal articles employing xenografting (Table 2; Buckingham et al., 2011; Campbell et al., 2012, 2015). The use of mice lacking an intact immune system, e.g., SCID mice, in investigations of GBM-associated epileptogenicity is controversial as the neuroinflammatory environment created and maintained by the tumor is a key contributor to the development of network hyperexcitability. As the primary aim of using animal models is to accurately recapitulate the disease as seen in humans, it stands to reason that GBM should be modeled in an immunocompetent animal to incorporate all aspects of the immune system that may play any role in seizure generation or GBM progression.

**Table 2:** Seminal experimental studies, with a primary objective of GBM-associated epilepsy investigation.

**Study**	**Model type**	**Time course**	**Chronic EEG?**	**Seizure frequency**	**Semiology**	**Spreading depolarizations?**	**Additional imaging?**
Köhling et al. (2006)	Allograft transplant: C6 cells, Wistar rats	nd	Y	nd	EEG: spike wave discharges	nd	N
Buckingham et al. (2011)	PDX transplant into SCID mice	nd	Y	*In vivo*: Average 2.5–6.5 events/hour	EEG: spontaneous epileptiform activity. Freezing behavior, facial automatisms, head tremor	nd	N
Campbell et al. (2012)	PDX transplant into SCID mice	21 days	N (*in vitro* only)	*In vitro*: 3.7 ± 0.6 events/min	nd	nd	N
Campbell et al. (2015)	PDX transplant into SCID mice	14–28 days	Y	*In vivo*:1 ± 0.3 events/day	EEG: spontaneous epileptiform activity. Freezing behavior, facial automatisms, head tremor	nd	N
MacKenzie et al. (2016)	Xenograft transplant: C6 cells, nude mice	21 days	Y	*In vivo*:11.0–18.7 events/week	EEG: spontaneous epileptiform activity.	nd	N
Bouckaert et al. (2020)	Allograft transplant: F98 cells, Fischer rats	21 days	Y	nd	EEG: spontaneous epileptiform activity. Ranging from non-convulsive to GTC seizures.	Y: seen in filtered trace, not acknowledged	Y—MRI
Hatcher et al. (2020)	Genetic: CRISPR Cas9-based IUE (deletion of Pten, Trp53, Nf1)	80 days	Y	*In vivo*: Day 80: 3.7–63.6 events/hour	EEG: spontaneous epileptiform activity, i.e., cortical spike discharges. GTC seizures (day 80).	Y	Y—Calcium imaging

*PDX, patient-derived xenograft; IUE, in-utero electroporation; EEG, electroencephalography; GTC, generalized tonic clonic; nd, not disclosed*.

The paucity of the literature including seizures as a measured variable in oncological studies is a substantial obstacle to collating information about mechanisms underlying GBM-related epileptogenesis. Where the experimental focus is oncogenesis or novel treatment development, the presence of seizures is often overlooked or only mentioned as a general indicator of a humane endpoint. Experimental animals typically express complex or simple focal seizures characterized by subtle behavioral changes such as freezing periods, facial automatisms, head tremors, or exaggerated startle responses to audiogenic stimuli (Köhling et al., 2006; Buckingham et al., 2011; Campbell et al., 2015). These events are completely spontaneous and may never occur in the presence of the researcher. Therefore, seizures could be easily missed if their investigation is not an objective of the study. However, arguably, neglecting to include seizures as a monitored variable in therapeutic studies has implications for clinical translation. The network changes occurring secondary to seizures and SDs in GBM undoubtedly impact tumor growth, disease progression, and potential risk of recurrence. Testing novel treatments in a model of GBM that does not account for seizure presence may confound results by omitting to include a potentially important influencer of therapeutic efficacy that affects a substantial portion of the patient population. Examples of how clinically-used anti-tumor treatments affect seizures in GBM have been examined in clinical trials (Chalifoux and Elisevich, 1997; Climans et al., 2020), but the literature primarily focuses on low-grade gliomas.

The Stupp regimen is the standard of care for GBM, consisting of resection followed 6 weeks later by post-surgical ionizing radiotherapy (RT) with concomitant TMZ chemotherapy and maintained by 20 weeks of adjuvant TMZ (Stupp et al., 2005, 2009; Fernandes et al., 2017). RT induces an acute inflammatory reaction to mediate its anti-tumor effects but, as it targets the vulnerable peri-cavity environment, this may paradoxically contribute to network hyperexcitability (Baskar et al., 2014). RT has been historically associated with improved seizure control in low-grade gliomas (Chalifoux and Elisevich, 1997; Rudà et al., 2013). However, extensive investigations have not yet been reported for GBM and therefore, seizure outcomes may differ. A prospective interventional trial evaluating the effect of RT on seizure activity in high grade (IV) gliomas is currently ongoing (Rades et al., 2021). Results from this study will aid understanding and inform future decisions pertaining to the application of individual anti-tumorigenic treatments in GBM patients with seizures. TMZ is an oral alkylating chemotherapeutic that combats tumor growth and progression through DNA methylation and cell cycle arrest. The efficacy of TMZ in GBM patients is correlated with increased methylation of the MGMT promoter (Perry et al., 2017). Interestingly this has also been associated with increased post-operative seizure control in one clinical study (Feyissa et al., 2019). This appears to indicate that increased susceptibility to TMZ treatment is associated with greater seizure control and better survival outcomes. A reduction in seizure burden in TMZ-treated MGMT-methylated epileptogenic GBM patients confers a proportional decrease in the activity of associated epileptogenic downstream signaling cascades. These pathways are known to encourage tumor growth, and so their interruption would restrict the ability of enduring tumor cells to progress into fully-fledged recurrent GBMs. However, other recent evidence suggests that the addition of TMZ to RT has a minimal effect on seizure presence in elderly GBM patients vs. RT alone (Climans et al., 2020). Climans et al. (2020) also demonstrated that decreased overall survival was significantly associated with seizures. This paradoxically supports the hypothesis that continuous seizure presence and associated excitotoxicity may increase the likelihood of tumor recurrence, and subsequently decrease the overall survival of epileptogenic GBM patients relative to patients without seizures. However, these conclusions were derived from previously acquired clinical trial data and MGMT methylation nor IDH-status were not taken into account in the reanalysis. Moreover, seizure outcomes were not reliably recorded in the original study potentially confounding results (Perry et al., 2017; Climans et al., 2020).

### Anti-Seizure Medication in GBM

The prevalence of seizures in GBM necessitates the use of anti-seizure drugs (ASDs) as the constant presence of the tumor increases the likelihood of seizure recurrence. GBM-associated seizures present unique challenges as they are often poorly controlled, requiring poly-therapy. However, there are several potential contraindications related to the use of ASDs in GBM, including increased risk of enhanced toxicity and undesirable interactions with anti-cancer treatments. Generally, non-enzyme inducing agents are first-line ASD options, i.e., levetiracetam (LEV), as they do not interfere with anti-tumor treatment strategies (Rosati et al., 2010; Kerkhof et al., 2013). Experimental evidence indicates that certain ASDs may have anti-tumorigenic effects, supporting a connection between seizures and mortality in GBM. Historically, valproic acid (VPA) treatment has been associated with a potential survival benefit in GBM patients (Kerkhof et al., 2013; Krauze et al., 2015; Lu et al., 2018). VPA is employed as an ASD as it disrupts GABA degradation and blocks voltage-gated Na^+^ and Ca^2+^ channels. Its anti-tumor effects are mediated through its actions as a histone deacetylase (HDAC) inhibitor and potentiation of the cytotoxicity induced by temozolomide (TMZ; Van Nifterik et al., 2012). Application of VPA to a variety of, but not all, GBM cell lines slowed cell cycle progression and inhibits proliferation (Knüpfer et al., 1998; Chavez-Blanco et al., 2006). However, the beneficial effect of VPA on GBM progression in the clinic is extremely variable. A recent study inferred that wild-type p53 status is necessary for VPA to enhance the anti-neoplastic effect of TMZ, demonstrating a potential reason for inconsistencies seen within the literature (Tsai et al., 2021).

In recent years, standards of care have shifted to favor LEV for anti-seizure treatment in GBM, and this has its own association with anti-cancer effects (Roh et al., 2020). It is currently favored as a therapeutic dose that can be achieved quickly in a clinic and there is a low likelihood of myelosuppression, hepatotoxicity, or hyponatremia (Hovinga, 2001). LEV binds to synaptic vesicle protein 2A, interfering with neurotransmitter release and reducing epileptogenic excitatory neurotransmission (Lynch et al., 2004). Evidence also indicates that LEV exerts a significant inhibitory effect on *O*^6^-methylguanine-DNA methyltransferase (MGMT) in GBM cell lines, conferring reduced resistance to TMZ (Bobustuc et al., 2010). LEV also increases MGMT transcription in normal astrocytes exhibiting a neuroprotective effect (Bobustuc et al., 2010). LEV treatment concurrent with standard chemo-radiation protocol has been associated with increased overall survival in *IDH-wildtype* GBM patients (Roh et al., 2020; Pallud et al., 2022).

A third-generation ASD, perampanel (PER), recently gained attention as it was shown to significantly reduce the seizure frequency of previously drug-refractory GBM-related seizures (Vecht et al., 2017; Dunn-Pirio et al., 2018; Izumoto et al., 2018; Coppola et al., 2020; Maschio et al., 2020). Its anti-seizure effects are mediated by its actions as a non-competitive AMPARs antagonist (Hanada et al., 2011; Frampton, 2015; Augustin et al., 2018; Brito da Silva et al., 2021). PER exhibits significant anti-proliferative effects on multiple GBM cell lines at various concentrations, and appears to reduce the overall concentration of extracellular glutamate (Lange et al., 2019; Salmaggi et al., 2021). Initial evidence suggests a correlation between PER treatment and tumor volume reduction on MRI-FLAIR (Izumoto et al., 2018). However, more clinical investigations are required before conclusions can be drawn as to the full extent of PER’s anti-tumor effects and any synergistic interactions it has with chemo-radiation regimens. Notably, PER is associated with various serious adverse effects, primarily psychiatric changes that are often the primary cause of discontinuation due to their prevalence (Rohracher et al., 2018; Villanueva et al., 2022). Administration of PER concomitant with anti-tumorigenic treatment regimens in a patient population already vulnerable to psychiatric changes, secondary to the GBM, must be carefully considered as adverse effects may be more frequent or severe.

It is of note that ASDs are not disease-modifying drugs, they do not prevent epileptogenesis but do reduce seizure burden. To our knowledge there are no known treatments that act to prevent epileptogenesis in the context of GBM, however, please see Terrone et al. (2020) for a comprehensive discussion of this work in other types of epilepsy.

### Novel GBM Therapeutics and Their Impact on Seizure Likelihood

Despite a wide research field dedicated to anti-GBM therapeutics, no significant positive advances have been made in prognosis for over a decade. As such, novel immunotherapeutic approaches such as immune checkpoint inhibitors (ICI), chimeric antigen receptor T cell (CAR-T) therapy, myeloid-targeted therapies, and tumor vaccines, have been the focus of GBM literature in recent years. GBM mediates an immunosuppressive TME by upregulating the expression of immune checkpoint molecules to reduce T cell activation and proliferation. This confers an almost intrinsic resistance to traditional anti-cancer treatments, making immune checkpoint molecules attractive therapeutic targets. In particular, increased expression of CTLA-4 is seen on Tregs and cytotoxic T lymphocytes, and PD-L1 on tumor cells and TAMs (Yu and Quail, 2021). Correspondingly, initial *in vivo* evidence demonstrated that the anti-PD-1 ICI nivolumab was remarkably effective at eradicating GL261 tumors. However, whilst essentially dominating the preclinical ICI research field, nivolumab’s effect failed to translate clinically (Yu and Quail, 2021). Interestingly seizures were the second most common neurological adverse event observed in clinical trials (Lim et al., 2017). ICIs provoke an adaptive immune response in the peri-cavity area to increase the activity and infiltration of cytotoxic T cells and pro-inflammatory cytokine secretion. As such, adverse effects have been attributed to “overshooting” T cell activation against antigens expressed on surrounding non-neoplastic cells (Roth et al., 2021). This mistargeting could act to promote hyperexcitability in peri-cavity neuronal networks post-resection, the time period at which systemic treatments are typically employed. Interestingly, an adaptive immune response characterized by the infiltration of cytotoxic CD8^+^ T cells and associated pro-inflammatory cytokine secretion is known to give rise to neuronal cell death and seizure induction in Rasmussen Encephalitis (Varadkar et al., 2014). Furthermore, the epileptogenic potential of ICIs is evidenced in reports of paraneoplastic encephalitis, and associated seizures, directly linked to the administration of anti-PD-1 (Shi et al., 2020) and anti-CTLA-4 (ipilimumab; Roth et al., 2021). It stands to reason that if a potentially epileptogenic ICI is employed in a seizure-vulnerable patient population, e.g., GBM, this may indirectly encourage tumor progression secondary to increased epileptiform activity and paradoxically result in decreased therapeutic efficacy. Unfortunately, there are no reports of the impact of ICIs on seizure presence and how this affects outcomes in GBM specifically. Therefore, the use of particular ICIs in the treatment of epileptogenic GBMs should be further investigated preclinically to validate their safe use in the clinic.

CAR-T therapy is another T-cell mediated immunotherapy, wherein a patient’s own T cells are harvested and engineered *ex vivo* to express chimeric antigen receptors (CARs) that recognize an identified tumor antigen, e.g., EGFRvIII, before being re-transplanted (Sterner and Sterner, 2021). CAR-T therapy application in GBM presents significant challenges namely due to intratumoral heterogeneity in antigen expression and the high likelihood of target antigens also being expressed in normal tissue (Maggs et al., 2021). Pathological overactive T-cell responses in CAR-T therapy commonly manifest as seizure-associated toxicities such as cytokine release syndrome (CRS) or immune effector cell-associated neurotoxicity syndrome (ICANS; Shimabukuro-Vornhagen et al., 2018). Headaches, hallucinations, hemiparesis, and seizures are some of the most prominent behavioral manifestations observed when CRS and ICANS occur secondary to brain-targeted CAR-T therapy (Shimabukuro-Vornhagen et al., 2018). The release of IL-1, TNF-α, IFN-β, and IL-6 are postulated to play key roles in both CRS and ICANS pathophysiology as they trigger an almost uncontrolled activation of microglia (Shimabukuro-Vornhagen et al., 2018; Siegler and Kenderian, 2020). In ICANS, activated CAR-T cells also provoke the release of pro-inflammatory cytokines IL-1 and IL-6, which subsequently activate endothelial cells of the BBB to drive its breakdown. The potentiation of significant neuroinflammation in peri-cavity tissue, when coupled with BBB breakdown, mirrors an environment seen in GBM-associated seizure generation. Furthermore, CAR-T cells can remain in circulation for a prolonged time post-infusion, and so continued downstream pro-inflammatory signaling may progressively encourage epileptogenesis in the vulnerable peri-cavity area. Whilst CRS/ICANS and the acute neurological toxicity associated with them are predominantly reversible, the potentiation of epileptogenic processes has permanent consequences and may result in paradoxical resistance to therapy secondary to seizure-potentiated tumor growth.

The therapeutic potential of macrophage reprogramming/repolarization has attracted a great deal of interest in recent years, but it also represents an area of uncertainty in its unknown relationship to epileptogenesis. The majority of TAMs are monocyte-derived macrophages (MDMs) that infiltrate the brain through the BBB and they account for up to 50% of the tumor mass (Parmigiani et al., 2021). M1-polarized macrophages display primarily pro-inflammatory, anti-tumor attributes making them an attractive therapeutic target. Whereas M2-polarized macrophages exhibit predominantly anti-inflammatory features with pro-tumor effects and are the subtype primarily associated with the GBM TME (Ma et al., 2021). Repolarization of TAMs within the TME focuses on therapeutic pro-inflammatory actions either in the tumor proper, in the absence of resection, or in peri-cavity tissue, negating the need for systemic application. However, this may have implications for epileptogenic GBMs as this area is highly vulnerable to the inflammation-induced alterations in NMDARs, AMPARs, and ion homeostasis that promote hyperexcitability. The anti-tumor consequences of macrophage repolarization are mediated through increased production of inducible nitric oxide synthase (iNOS), TNF-α, IL-1β, IL-6, and chemokines associated with cytotoxic T cell recruitment (Duan and Luo, 2021). As previously stated, these are all mediators known to contribute to epileptogenesis. Whilst there are no reported investigations into macrophage reprogramming in epileptogenic GBMs, studies have acknowledged seizures as a common side effect in cytosine-guanine oligonucleotide (CpG-ODN) mediated MDM repolarization (Carpentier et al., 2006, 2010). Intracranial infusion of litenimod (CpG-28) in a cohort of 34 recurrent GBM patients was associated with seizures of either generalized or focal semiology in 14 patients (Carpentier et al., 2010). The downstream actions of M1-polarized macrophages and aforementioned evidence suggests that TAM reprogramming may possess at least an acute epileptogenic capability, and indicates a clear need for further preclinical investigation.

Whilst it is clearly important that seizure presence and prevalence be a monitored variable in preclinical studies of novel anti-tumorigenic treatment regimens, it ultimately returns to a balance of risk-benefit when translating these therapeutics into the clinic. If these therapies effectively eradicate the GBM, despite increasing the risk of epilepsy, then this would be tolerable. However, if these therapies only extend survival by a few months and this time is associated with debilitating seizures, would this still be an acceptable outcome?

### Surgical Resection and Recurrence

**Figure 3:**
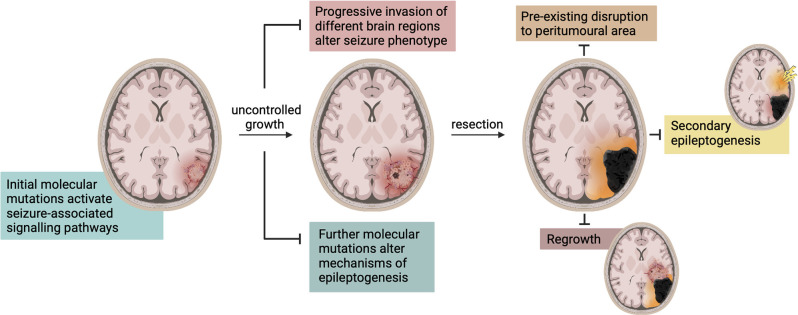
Changes in seizure characteristics during disease progression and treatment. There are a multitude of mechanisms that alter seizure characteristics and phenotype in GBM. The molecular mutations and consequently activated signaling pathways change throughout its progression and as it enters new areas of the brain. Upon resection, these mechanisms are altered again and often accompanied by a period of seizure cessation. Seizures may then be produced in the peri-cavity region due to pre-existing network disruption or tumor regrowth, or in an entirely different region due to secondary epileptogenesis.

Another equally important challenge in preclinical research is the focus on pre-resective models of GBM, all current epileptological GBM research has been performed in pre-resection models. These represent a valuable method to investigate underlying mechanisms but may not be as clinically relevant. The majority of GBM patients are eligible for resective surgery to remove the bulk tumor, and as a corollary, the most common clinical scenario involves a post-resection brain. The post-resection environment demonstrates a substantially altered neural network, even vs. pre-resection, and as such the mechanisms of hyperexcitability and seizure generation may also be altered (Figure 3). Resection models of GBM have been developed (Kauer et al., 2011; Momiyama et al., 2013; Bianco et al., 2017; Otvos et al., 2021; Tang et al., 2021), but their use has not been reported in the investigation of GBM-related epileptogenesis. Therefore, there is currently no evidence indicative of how mechanisms of excitability and seizure generation are altered in a post-resection brain. Both pre-resective and recurrent GBMs display considerable intratumoral heterogeneity, but recurrent GBMs may also have a slightly different molecular phenotype than the tumor it originated from (Campos et al., 2016). The recurrent tumor acquires new genetic aberrations as it grows (Kim et al., 2015), potentially resulting in different reactions to anti-tumor or anti-epileptic therapeutic regimens used to treat the pre-resective tumor. This has downstream effects on the ability to make research-informed decisions regarding anti-seizure treatment regimens post-resection in the clinic, and demonstrates an incomplete understanding of the impact seizures have on disease progression.

The recurrence of seizures following GBM resection could be attributed to a phenomenon known as secondary epileptogenesis (Figure 3), wherein an extended epileptogenic network enables epileptogenesis to continue following the removal of a primary focus (Morrell et al., 1956; Morrell, 1985; Scholly et al., 2017). Morrell (1985) defined secondary epileptogenesis as a three-stage process in which an actively discharging epileptogenic region gradually induces paroxysmal activity in the cellular components of distally connected normal neuronal network(s). In this context, GBM can be considered the primary epileptogenic foci and the generation of seizures is predominantly dependent on its presence (stage 1). Following GBM resection, or removal of the primary focus, there is a period of temporary seizure cessation whilst the network re-adjusts to further reorganization, aligning with stage 2. Finally, consistent with stage 3, underlying epileptiform activity in a secondary previously-connected focus persists independently to manifest as post-resection seizures. This is a hypothesis not previously considered in experimental or clinical literature, and evidently the controversial existence of secondary epileptogenesis requires greater attention in the context of GBM.

New-onset seizures or their recurrence post-tumor resection is common, and have been independently associated with tumor recurrence, but evidence also suggests a correlation to the extent of resection (EOR). Generally, surgical treatment of GBM can range from a minimally invasive surgical biopsy to a craniotomy involving varying degrees of “total” tumor resection (McGirt et al., 2009; Brown et al., 2016; Tan et al., 2020). Increased EOR is independently associated with increased survival rates (McGirt et al., 2009), and this also correlates with seizure freedom. Patients who have undergone gross total tumor resection (GTR; >80%) are much more likely to be seizure-free post-operatively than sub-total resection (SubTR; <70%; Liang et al., 2016). Still significantly higher rates of seizure freedom are seen with supra-total resection (SupraTR; >100%) in the form of anterior temporal lobectomy (ATL; Borger et al., 2021). Comprehensive investigations into the effect of seizures on risk of tumor recurrence and how this is impacted by EOR have not yet been reported. However, interesting initial evidence from a medium-sized cohort study appears to indicate that patients with post-operative seizures may exhibit less time to tumor recurrence vs. those without, independent of EOR (Liang et al., 2016). It is well recognized that the peri-tumoral area is a primary source of epileptiform activity and hyperexcitability, as previously discussed. It has been postulated that, as this area often goes un-resected, post-resection seizures may continue to originate from the pre-existing epileptogenic locus in the original peri-tumoral border. The process of epileptogenesis and associated tumorigenic network alterations may potentially continue in this peri-cavity area even independent of tumor presence. Increased use of intraoperative electrocorticography (ECoG) in GBM patients with pre-operative seizure history has the potential to increase maximal resection margins to include greater portions of peri-tumoral epileptogenic loci. This would increase the likelihood of post-operative seizure freedom and potentially influence tumor recurrence risk.

**Figure 4:**
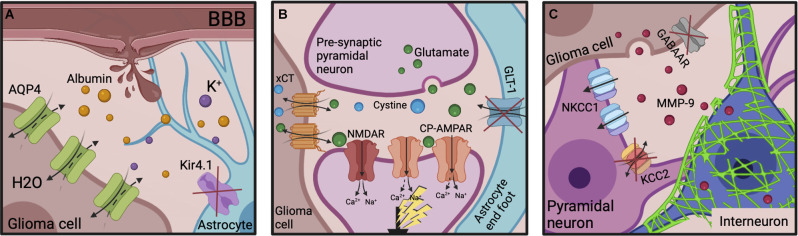
Epileptogenic molecular mechanisms post-GBM resection. **(A)** The neuroinflammatory environment and BBB disruption originally potentiated by the tumor can also be transiently exacerbated by the resective surgery itself. Serum albumin infiltration evokes Kir4.1 downregulation on reactive astrocytes, enabling K^+^ accumulation. AQP4 expression is increased on glioma cells, decreasing the ECS. **(B)** Increased xCT expression on glioma cells and decreased GLT-1 expression on astrocytes leads to extracellular glutamate accumulation in the peri-cavity region. It’s post-synaptic action at NMDARs and CP-AMPARs increases neuronal excitability. **(C)** The remaining glioma cells continue to release MMP-9 which induces PNN degradation. GABA_A_R expression is almost absent on glioma cells to aid their proliferation. NKCC1 is upregulated and KCC2 downregulated on surrounding pyramidal neurons, reversing the Cl^−^ gradient. The subsequent decreased inhibitory influence disinhibits pyramidal neuronal firing enabling seizure generation. ECS, extracellular space; BBB, blood-brain barrier; NMDAR, NMDA receptor; CP-AMPARs, Ca^2+^-permeable AMPARs; MMPs, matrix metalloproteinases; PNNs, perineuronal nets.

Additionally, the cycle of epileptogenesis may be potentiated post-resection by the small populations of neoplastic cells that invariably persist in the “normal” parenchyma surrounding the resection cavity. These remaining glioma cells promote network reorganization of the peri-cavity area through further BBB breakdown (Figure 4A), and continued dysfunctional glutamatergic (Figure 4B), and GABAergic signaling (Figure 4C). Additionally, their release of MMP-9 works to degrade FS-PV+PNNs (Figure 4C). Therefore, the hyperexcitable peri-cavity area in epileptogenic GBM patients is highly likely to actively be undergoing epileptogenesis secondary to the actions of these enduring glioma cells. These seizures- and glioma cell-driven aberrances will result in further progressive reorganization of peri-cavity neuronal networks, working in pathological synergy to promote recurrence. The multiple month latency to seizure generation and tumor recurrence following initial GBM resection appears to fit with this timeline (Liang et al., 2016). It is pertinent to mention that the cumulative risk of *de novo* post-operative epilepsy is generally increased following all craniotomy procedures, but this is markedly higher in patients undergoing craniotomies for GBM resection (13.9% vs. 23.6% respectively; Giraldi et al., 2021).

### Novel Technologies for Electrophysiological Investigation in GBM

By nature, the seizures that occur in models of GBM-related epilepsy are spontaneous and so represent unique challenges. A comprehensive investigation of epileptogenesis in GBM models requires continuous electrophysiological monitoring of epileptiform activity. This is typically performed through chronic implantation of electroencephalographic devices, as it is impossible to predict seizure occurrence without pro-convulsant intervention by pharmaceutical or stimulatory means. Furthermore, the lack of overt behavioral manifestations of seizure presence in experimental animals renders video monitoring insufficient in the absence of accompanying EEG recordings. Equipment to record epileptiform activity long-term is widely accessible and readily employed in experimental epilepsy studies, commonly as tethered recordings or subcutaneous battery implantation to power the recording headstage (Wykes et al., 2012). However, these technologies are largely unsuitable for use in a progressive GBM model as they primarily employ a single or few electrodes, rather than configurations that allow high-density mapping. If a rigid electrode is invasively implanted into the brain, this could result in mechanical and physical damage secondary to the tumor growth, and a progressive attenuation of the signal as the recording site is enveloped by the tumor. Therefore, to effectively monitor epileptiform activity in GBM, any electrophysiological methods must include some form of multi-electrode array to account for the progressive expansion of the peri-tumoral border. Furthermore, whilst some electrophysiological techniques, i.e., epicortical arrays, can be used in conjunction with imaging techniques, conventional electrodes are poorly suited due to the presence of overwhelming artifact or magnetic interference with electronics. This represents an obstacle in current preclinical research, wherein the restricted ability to employ live imaging methods in conjunction with ECoG monitoring hinders the investigation of seizures and SDs in GBM.

The ideal preclinical scenario would incorporate MRI-compatible multi-electrode, flexible, penetrating arrays that can be placed at the peri-tumoral border, and are capable of electrographically mapping the changes to network excitability associated with progressive tumor growth over multiple areas of the brain. The closest preclinical example of this is seen in Hatcher et al. (2020) wherein *in vivo* Ca^2+^ fluorescence imaging was used in conjunction with EEG monitoring in a CRISPR-Cas9 IUE model of GBM-associated epilepsy, with great success. However, Ca^2+^ imaging is a complex technique not readily adopted nor clinically translatable. This hurdle presents challenges in both transplantation and genetic models of GBM-related epilepsy as tumor volume and growth rates can vary between animals. As a corollary, previous conclusions reached about the causal relationship between volume or growth patterns and the development of epileptiform activity are more illustrative than certain statements.

The presence of SDs in GBM-associated epilepsy further complicates electrophysiological investigations. The massive, abrupt change in activity seen in SD results in cortical electrical potential changes over a wide spectral range. This primarily manifests as slow potentials approaching the DC (or 0 Hz) level. Negative potential shift is an important identifier of SD but is not measured using standard AC-coupled (>0.5 Hz) highpass filtered electrophysiological methods. This can be partially overcome by the use of amplifiers capable of recording at lower high-pass filter limits (>0.02 Hz), or by using a DC-coupled system. However, substantial attenuation of the amplitude and distortion of the SD waveform is evident when using passive metal-based electrodes, as well as issues with drift and amplifier saturation. The presence of SD has not yet been investigated intra- or post-operatively in GBM patients, epileptogenic or otherwise, and only very few experimental studies acknowledge its presence (Bouckaert et al., 2020; Hatcher et al., 2020). Current detection of SDs in humans is mediated using platinum electrodes. They allow for the unfiltered recording of DC shifts and have a relatively high signal-to-noise ratio (SNR), but the unfortunate presence of artifacts and transients makes them unsuitable for accurate monitoring and mapping of SD in GBM. Experimental studies employ solution-filled glass micropipettes and silver or silver chloride (Ag/AgCl) wires to detect SDs (Hatcher et al., 2020), but these offer little to no spatial resolution. Additionally, the inherent toxicity of Ag/AgCl makes it inappropriate for use in clinical studies.

There is a clear need for novel means of electrophysiological recording that can sustain continuous and simultaneous recording of both high frequency synchronous epileptiform activity and SD DC-shifts across the brain. Importantly, these technologies need to be compatible with a variety of imaging modalities in order to draw direct conclusions relating to the interaction between tumor presence and seizure generation. The application of novel technologies currently in development for preclinical investigations of epileptogenesis in GBM are crucial to improving our understanding. One such example of which is graphene-based solution-gated field-effect transistors (gSGFETs). gSGFETs have the potential to fill this technology gap as they offer the means to record SDs, either independently or concurrently with high-frequency activity, both epi- and intra-cortically with high spatiotemporal fidelity (Hébert et al., 2018; Masvidal-Codina et al., 2019, 2021; Bonaccini Calia et al., 2021). The potential for SDs to exacerbate tissue damage and promote mechanisms of hyperexcitability in GBM could have consequences for disease progression, prognosis, and resistance to therapies.

## Discussion

It is clear that GBMs create a pro-epileptogenic environment *via* a multitude of mechanisms, and thus the associated seizures present unique clinical challenges. However, it is also known that some GBM patients do not present with, and never experience tumor-associated seizures or epilepsy. Therefore, we may gain more mechanistic insight into what the important essential factors are for the development of epilepsy by understanding why some patients do not develop epilepsy despite an aggressive tumor environment.

The answer may lie somewhere in the complex interaction between genetic influences and acquired mechanisms of epilepsy. There is a precedent of diseases that are associated with epilepsy where seizures manifest only following treatment. In neurocysticercosis, intracranial cysts evade detection by the immune system through various mechanisms, and it is only once they are treated that seizures are generated (Carpio et al., 1998; Prodjinotho et al., 2020; Espino et al., 2022). This master evasion of the immune system is an aspect mirrored in GBM. In the GBM TME of some patients, the tumor may promote such extensive anti-inflammatory signaling to suppress the immune system, that it indirectly transiently discourages epileptogenesis. This may also offer a further potential explanation for new-onset seizures post-resection. Wherein the removal of the tumor may provoke such an aggressive inflammatory immune response that it potentiates seizure generation.

Recent experimental evidence suggests that tumor-localized expansion of certain pathological glioma subpopulations enriched for epilepsy-associated genes may confer hyperexcitability secondary to neosynaptogenesis (Lin et al., 2017). Therefore, if the proportional expression of certain glioma subpopulations in a tumor differ to not favor this particular population, then the changes incurred by the aforementioned peri-tumoral mechanisms may not be cumulatively sufficient to evoke hyperexcitability and ultimately initiate epileptogenesis. This is a link between genetic and acquired mechanisms of GBM-associated epilepsy not previously delineated (Lin et al., 2017). The intratumoral heterogeneity of GBM, and its unique microenvironment, support this theory. This is indicative that many different pro-epileptogenic mechanisms must work in pathological synergy in order for seizures to occur. Further investigations are needed to unravel the interactions between genetic predisposition and acquired mechanisms of epilepsy in GBM as this is clearly an area that could provide great insight into the determinants of seizure presence in a population of GBM patients. Investigations in this vein would also support the transition to more personalized therapeutics, as is an important current area of research in GBM.

Ultimately, a better understanding of the relationship between GBM, epilepsy, and SDs will stem from further experimental investigations. Whilst clinical studies have provided initial evidence connecting tumor location to seizure presence, the reluctance of oncology studies to stray from the standard striatal injection site precludes further preclinical investigation. Incorporating a variety of injection sites, i.e., mesial temporal or frontal lobe structures, into implantation models would help to further our understanding of how GBM growth in different brain regions predisposes a patient to comorbidities. Different areas of the brain are considerably different in how their networks function. Therefore, certain networks may communicate in ways that have a more favorable influence on the relationship between tumor growth and secondary epileptogenesis. Greater insight into how tumor location specifically affects the process of epileptogenesis will undoubtedly have clinical significance. Patients identified as predisposed to developing seizures may receive slightly altered anti-tumorigenic regimens that also simultaneously address the underlying network dysfunction occurring during tumor-associated epileptogenesis, instead of just using an adjuvant ASD.

It is important to emphasize that novel technologies need to be developed to comprehensively investigate the impact tumor location has on epileptogenesis. The ability to map seizure and SD propagation across multiple brain areas with high fidelity is an aspect of electrophysiology that has eluded the research community up until very recently. Initial experimental evidence shows that cortical involvement is progressive in preclinical GBM-epilepsy models (Hatcher et al., 2020). Therefore, the availability of both epi-dural and intra-cortical arrays is crucial to spatially map the relationship of epileptiform activity with the expansion of the peri-tumoral border. Evidently, the spontaneous nature of epileptiform activity and SDs in this disease model necessitates 24/7 electrophysiological recording. However, chronic monitoring is a substantial experimental and technological investment and therefore is not readily performed. The inaccessibility, and unavailability, of this technology is a major obstacle to investigating seizures as a measured variable in oncology studies. Many novel anti-tumorigenic treatments appear to have pro-epileptogenic potential, but it is only with increased accessibility of electrophysiological technologies that seizures will be included and monitored in these studies. The field of GBM research overwhelmingly focuses on the development of novel treatment options. Therefore, it is crucially important that the effect seizures and SDs may have on therapeutic efficacy is evaluated to best inform their clinical success.

## Author Contributions

KH, KK, and RW developed the concept and designed the initial manuscript. KH wrote the manuscript and produced the figures. RW and KK assisted with manuscript revisions. All authors contributed to the article and approved the submitted version.

## Conflict of Interest

KK would like to declare he is co-founder and holds equity in INBRAIN Neuroelectronics (Barcelona, Spain). The remaining authors declare that the research was conducted in the absence of any commercial or financial relationships that could be construed as a potential conflict of interest.

## Publisher’s Note

All claims expressed in this article are solely those of the authors and do not necessarily represent those of their affiliated organizations, or those of the publisher, the editors and the reviewers. Any product that may be evaluated in this article, or claim that may be made by its manufacturer, is not guaranteed or endorsed by the publisher.
